# Characteristics of patients with depression initiating or switching antidepressant treatment: baseline analyses of the PERFORM cohort study

**DOI:** 10.1186/s12888-018-1657-3

**Published:** 2018-03-27

**Authors:** Josep Maria Haro, François-Xavier Lamy, Bengt Jönsson, Martin Knapp, Mélanie Brignone, Hugo Caillou, Ylana Chalem, Lene Hammer-Helmich, Benoît Rive, Delphine Saragoussi

**Affiliations:** 1 Parc Sanitari Sant Joan de Deu, Universitat de Barcelona, CIBERSAM, C/ Doctor Antoni Pujadas 42, 08830 Sant Boi de Llobregat, Barcelona, Spain; 2 Lundbeck SAS, 37-45 Quai du Président Roosevelt, 92445 Issy-les-Moulineaux, France; 3 Department of Economics, Stockholm School of Economics, Sveavägen 65, 113 83 Stockholm, Sweden; 4 Personal Social Services Research Unit, Cowdray House, London School of Economics and Political Science, Houghton Street, London, WC2A 2AE UK; 5 Inferential, 35 rue Godot de Mauroy, 75009 Paris, France; 6 H. Lundbeck A/S, Ottiliavej 9, 2500 Valby, Denmark; 7 Present address: Merck KGaA, Darmstadt, Germany; 8 Present address: Capionis, Bordeaux, France; 9 Present address: Pierre Fabre SA, Boulogne, France; 10 Present address: Janssen, Issy-les-Moulineaux, France

**Keywords:** Depression, Drug switching, Observational study, Baseline survey

## Abstract

**Background:**

Patients who require a switch in their antidepressant therapy may have different clinical profiles and treatment needs compared with patients initiating or maintaining a first-line antidepressant therapy.

**Methods:**

The Prospective Epidemiological Research on Functioning Outcomes Related to Major depressive disorder (MDD) (PERFORM) study was a 2-year observational cohort study in outpatients with MDD in five European countries. Enrolled patients were either initiating or undergoing the first switch to an antidepressant monotherapy. Baseline data on patients’ clinical status, functioning, productivity, quality of life and medical-resource use were compared in a cross-sectional baseline analysis.

**Results:**

A total of 1402 patients were enrolled, of whom 1159 (82.7%) provided analysable baseline data. The majority (78.7%) of the analysable population were initiating antidepressant treatment and most (83.6%) were enrolled and followed up by general practitioners. Compared with patients initiating antidepressants, those switching antidepressants (21.3%) tended to have more severe depressive symptoms, greater anxiety, worse health-related quality of life, greater functional impairment, greater medical-resource use and had a different medical history. Limitations included an over-representation of switches due to lack of efficacy among patients who were switching treatment, as patients were selected based on presence of depressive symptoms.

**Conclusions:**

Patients with MDD who are switching treatment for the first time have a different profile and different depression-associated health needs compared with those initiating treatment. Therapeutic management should therefore be adapted for patients who switch.

**Trial registration:**

ClinicalTrials.gov NCT01427439; Retrospectively registered 26 August 2011.

**Electronic supplementary material:**

The online version of this article (10.1186/s12888-018-1657-3) contains supplementary material, which is available to authorized users.

## Background

Pharmacological therapies are commonly used in the management of patients with depression; however, a significant proportion of patients do not respond to an adequate trial of the first antidepressant prescribed in a depressive episode [1–3]. The Sequenced Treatment Alternatives to Relieve Depression (STAR*D) trial reported that up to 37% of patients initiating antidepressant treatment in clinical practice achieved remission with first-line treatment [2, 4]. Change of treatment is thus a frequent therapeutic action in patients with depression who do not respond to their initial treatment. Treatment guidelines recommend a range of pharmacotherapeutic approaches for patients who need a treatment change from their first-line treatment, including treatment switch (changing to a different antidepressant), combination therapy (adding a second antidepressant) and augmentation (adding an agent that is not generally considered an antidepressant, e.g. an antipsychotic) [5–8]. Switching therapy tends to be recommended if a patient experiences an inadequate response and/or tolerability problems, whereas combination and augmentation strategies would be used for patients who had experienced a partial response to the initial therapy but had residual symptoms and had not experienced tolerability problems [9]. Several studies have found that switching is a common choice in clinical practice for patients requiring second-line treatment, including those with an inadequate response to first-line antidepressant therapy [10–13]. Clinical evidence supports switching antidepressants in such patients [14, 15].

In considering a switch from first-line treatment, guidelines recommend taking into account whether the patient has experienced adverse effects and patient preference [5, 8]. However, some studies have shown that, in clinical practice, patients who switch antidepressants may have a very different clinical profile compared with those who do not switch treatment. An observational study based on data from the General Practice Research Database (GPRD; now known as the Clinical Practice Research Datalink) in the UK found that patients who switched antidepressants tended to have a more severe psychiatric profile compared with those who maintained their initial therapy [12]. In addition, differences in the degree of resolution for symptoms of occupational impairment have been noted between patients responding to first-line treatment and those responding to second-line treatment [16]. Such findings suggest that patients who require a switch in their antidepressant therapy may benefit from a more detailed clinical assessment of their illness and a specific management approach, distinct from that taken with patients who are initiating or maintaining a first-line antidepressant therapy.

The Prospective Epidemiological Research on Functioning Outcomes Related to Major depressive disorder (MDD) (PERFORM) study was designed to better understand the course of a depressive episode and its impact on patient functioning over a 2-year period in patients with MDD in clinical practice in five European countries. The study recruited patients with a diagnosis of MDD who were either initiating antidepressant monotherapy or who were switching antidepressant monotherapy for the first time*.* The aim of the cross-sectional baseline analyses presented in this paper was to describe the characteristics of patients who were initiating or switching antidepressant monotherapy when entering the PERFORM study and to compare their depression-related clinical profile, daily functioning, health-related quality of life and resource use.

## Methods

### Study design and patients

The PERFORM study is a 2-year observational cohort study in outpatients with MDD enrolled by either a primary care physician or a psychiatrist at 194 sites in five European countries (France, Germany, Spain, Sweden and the UK). In each country the study sites were selected to reflect the national proportions of these clinicians treating patients with depression. At enrolment, eligible patients were aged 18–65 years, had a current diagnosis of MDD according to the Diagnostic and Statistical Manual of Mental Disorders IV Text Revision (DSM-IV-TR), and were either initiating antidepressant monotherapy or were undergoing their first switch of antidepressant. Patients were excluded if they had schizophrenia or other psychotic disorders; bipolar disorder; substance dependence; dementia or other neurodegenerative disease significantly affecting cognitive functioning; or a mood disorder due to a general medical condition or substances. Patients receiving a combination of different antidepressant treatments at the time of the initial consultation were also excluded.

The physician’s choice of the drug used to treat each patient was based on clinical judgement alone and was not influenced by the decision to enter the study. All patients enrolled in the study provided written informed consent. The necessary ethical approvals were obtained for each study site before study initiation. The study was registered at ClinicalTrials.gov: NCT01427439 on 26 August 2011.

### Baseline characteristics

Patient characteristics recorded at baseline included demographic information; the characteristics of the current episode of depression and – for patients switching antidepressants – the main reason for switching, according to the physician; history of depression; and the presence of mental health disorders other than depression, and of any functional syndromes.

Clinical profile at baseline was assessed by all patients using the 9-item Patient Health Questionnaire (PHQ-9) [17] and by all participating investigators using the Clinical Global Impressions–Severity of illness scale (CGI-S) [18]. The Montgomery–Åsberg Depression Rating Scale (MADRS) [19] was also administered when recruitment was conducted in the specialized sector and patients were evaluated by psychiatrists. The PHQ-9 scores were used to categorize the severity of symptoms as “minimal” (score 0–4), “mild” (score 5–9), “moderate” (score 10–14), “moderately severe” (score 15–19) or “severe” (score 20–27) for analysis. The CGI-S responses were analysed by the category assigned by the physician on a 7-point scale ranging from 1 = not ill, to 7 = extremely ill. The MADRS responses were analysed by total score.

Daily functioning and quality of life were assessed using self-administered instruments. Functioning was evaluated using the Sheehan Disability Scale (SDS) [20] including three dimensions (work, social and family functioning). The “activity impairment” dimension of the Work Productivity and Activity Impairment questionnaire (WPAI) was also applied in order to evaluate functioning [21]. The other dimensions of the WPAI are not presented as they aim to measure productivity and are relevant only to the subpopulation in employment. The Arizona Sexual Experience Scale (ASEX; five items, each scored from 1 to 6) [22] was used to assess sexual functioning. Patients were categorized as having sexual dysfunction if their total ASEX score was ≥19 or their score was ≥5 for one item or ≥ 4 for at least three items. Patient-reported outcomes were also used to assess health-related quality of life using the physical and mental health dimensions of the Medical Outcomes Study Short-Form (12-item) Health Survey (SF-12) [23] and the EuroQol 5-Dimensions questionnaire (EQ-5D; in UK patients only) [24]. Utility scores were derived from the EQ-5D scales by applying UK tariffs [25].

Resource use during the 12 weeks before baseline was assessed by physician reporting of physician visits, hospitalizations and periods of sick leave. Resource-use data were reported for patients who had experienced a depressive episode of at least 8 weeks’ duration as: per cent of patients reporting at least one visit (physician or other healthcare professional) or 1 day of hospitalization or sick leave over the recall period; number of visits (physician or other healthcare professional) or days of hospitalization or sick leave for patients with at least one visit or day of the corresponding resource item; and overall number of visits (physician or other healthcare professional) or days of hospitalization or sick leave. The restriction of this analysis to patients with a minimum depressive-episode duration of 8 weeks was applied to allow an appropriate comparison between the two treatment groups, given the between-group difference in duration of the current depressive episode.

### Data analysis

Patients included in the analysable dataset were those who met the study selection criteria and completed a baseline assessment form as well as at least one post-baseline assessment or outcome questionnaire. Summary statistics (mean, standard deviation, median, minimum and maximum) were calculated for continuous variables, and numbers and percentages were calculated for categorical variables. Analyses were performed for both groups of patients: those switching and those initiating treatment. Differences between these groups were investigated using either Student’s *t* test for quantitative variables or chi-squared test (or Fisher’s exact test where appropriate) for qualitative variables. All analyses were univariate as the aim was to describe the characteristics of the study populations rather than to explain or identify any associations. Statistical significance was assumed at *p* < 0.05. Statistical analyses were performed using SAS® statistical software, Version 9.2 (SAS Institute, Cary, NC, USA).

## Results

### Subjects

A total of 1895 patients were screened, 1402 of whom were enrolled in the study. The first patient was screened on 25 February 2011 and the last patient completed the study on 19 February 2015. The most frequent reason for non-enrolment of screened patients (*n* = 493) was patient’s decision (*n* = 203; 41.2%). Of those who were enrolled, 1159 (82.7%) provided analysable data. Patients who were enrolled but not included in the analysable population (*n* = 243) were excluded because they violated at least one of the inclusion and/or exclusion criteria at baseline (*n* = 167) or they had not completed a post-baseline case report form or at least one post-baseline questionnaire in the predefined timeframe (*n* = 101, including 76 who met the inclusion criteria at baseline). The majority of patients were enrolled and followed up by general practitioners (*n* = 969; 83.6%). Overall the analysable population had a mean age of approximately 44 years, and the majority (*n* = 848; 73.2%) were female (Table 1). Included patients were from the UK (*n* = 341; 29.4%), France (*n* = 339; 29.2%), Spain (*n* = 270; 23.3%), Germany (*n* = 164; 14.2%) and Sweden (*n* = 45; 3.9%).

**Table 1:** Patient characteristics at baseline

Characteristic	Switching (*n* = 247)^a^	Initiating (*n* = 910)^a^	*P*-value: switching vs initiating	Total (*n* = 1159)^b^
Age, mean ± SD (years)	46.0 ± 11.7	43.8 ± 12.0	**0.012**	44.3 ± 12.0
Female (%)	76.1	72.5	0.259	73.2
Marital status (%)			**0.021**	
Single	20.6	21.9		21.6
Married/couple	54.7	59.6		58.5
Divorced/separated	18.6	16.2		16.7
Widowed	6.1	2.4		3.2
Education (%)			**< 0.001**	
No degree or diploma	8.1	3.5		4.5
Elementary school	32.8	22.1		24.4
High school	31.6	38.4		36.9
Non-university degree	13.0	14.3		14.0
University degree	14.6	21.8		20.2
Work status (%)				
Paid employment or self-employed	59.5	68.8	**0.006**	66.8
Unemployed	20.2	17.1	0.259	17.8

*Abbreviation: SD* standard deviation

*P*-values in bold indicate statistically significant differences

^a^Number of patients included in the between-group comparison; information regarding whether the patient was initiating or switching treatment was missing for two patients

^b^Total number of patients providing data

At baseline 910 (78.7%) patients were initiating antidepressant treatment and 247 (21.3%) were switching antidepressant for the first time; the treatment status of two patients was unknown. The main causes of switching (recorded for 216 patients) were lack of efficacy (*n* = 167; 77.3%), adverse events (*n* = 20; 9.3%), patient’s decision (*n* = 15; 6.9%) and lack of compliance (*n* = 7; 3.2%). Compared with patients who were initiating antidepressants, approximately twice as many patients in the analysable population who were switching antidepressants were being treated by psychiatrists at the time of enrolment (*n* = 124 vs 66, respectively; *P* < 0.001; Table 2).

**Table 2:** Medical profile, functioning and quality of life at baseline

	Switching (*n* analysed)^a^	Initiating (*n* analysed)^a^	*P*-value: switching vs initiating	Total^b^ (*n*)
Characteristics of current depressive episode				
Treating physician (% of patient group)	(247)	(910)	**< 0.001**	(1159)
General practitioner	73.3	86.4		83.6
Psychiatrist	26.7	13.6		16.4
Duration of episode (%)^c^	(247)	(910)	**< 0.001**	(1157)
< 1 week	0.4	1.9		1.6
1–2 weeks	2.8	6.7		5.9
2–4 weeks	14.6	23.2		21.3
4–8 weeks	17.4	20.4		19.8
> 8 weeks	64.8	47.8		51.4
Significant symptoms of anxiety (%)^d^	74.5 (247)	59.3 (910)	**< 0.001**	62.6 (1158)
Symptoms treated with anxiolytics (%)	53.1 (130)	38.6 (376)	**0.004**	42.2 (507)
Questionnaire scores, mean ± SD				
PHQ-9	18.4 ± 5.3 (198)	17.4 ± 5.3 (740)	**0.014**	17.6 ± 5.3 (940)
CGI-S	4.4 ± 1.0 (246)	4.1 ± 1.0 (908)	**< 0.001**	4.2 ± 1.0 (1155)
MADRS^e^	32.5 ± 7.1 (66)	32.9 ± 7.2 (124)	0.724	32.7 ± 7.1 (190)
SDS				
Total score	20.6 ± 6.6 (147)	18.9 ± 6.7 (601)	**0.004**	19.2 ± 6.8 (750)
Work/school disruption	6.7 ± 2.7 (150)	6.1 ± 2.8 (614)	**0.020**	6.3 ± 2.8 (766)
Social life/leisure activities’ disruption	7.0 ± 2.4 (179)	6.4 ± 2.5 (704)	**0.003**	6.6 ± 2.5 (885)
Family life/home duties’ disruption	7.0 ± 2.4 (179)	6.4 ± 2.5 (701)	**0.001**	6.5 ± 2.5 (882)
WPAI^f^	66.0 ± 23.5 (194)	59.9 ± 24.9 (740)	**0.002**	61.1 ± 24.8 (936)
ASEX				
Total score	22.2 ± 5.8 (169)	21.2 ± 5.7 (623)	**0.031**	21.4 ± 5.7 (793)
Sexual dysfunction (%)	84.6 (188)	80.3 (695)	0.182	81.1 (884)
SF-12	(190)	(720)		(912)
PCS	40.6 ± 11.4	46.4 ± 12.0	**< 0.001**	45.2 ± 12.1
MCS	26.8 ± 9.0	26.4 ± 9.2	0.598	26.5 ± 9.2
EQ-5D utility score^g^	0.5 ± 0.3 (48)	0.6 ± 0.3 (227)	**0.044**	0.5 ± 0.3 (276)
Other current illnesses				
Mental-health disorders other than depression (%)	(247)	(910)		(1159)
Alcohol abuse or dependence	1.6	3.0	0.245	2.7
Other abuse disorders	1.2	1.3	1.000	1.3
Somatoform disorders	10.5	7.0	0.069	7.9
Eating disorders (anorexia, bulimia)	10.5	7.5	0.119	8.1
Other	0.0	0.8	0.357	0.6
Functional syndromes (%)	(247)	(910)		(1159)
Chronic pain	23.9	14.7	**< 0.001**	16.7
Chronic fatigue	17.0	15.9	0.685	16.1
Fibromyalgia	13.0	5.2	**< 0.001**	6.8
Premenstrual syndrome	4.9	3.1	0.174	3.5
Sleep disorders	29.1	27.9	0.701	28.2
Other	2.4	2.9	0.716	2.8
Previous depressive episodes				
History of depression (%)				
Previous episode	72.0 (246)	52.4 (910)	**< 0.001**	56.6 (1157)
Episode within previous 12 months if previous episode	33.9 (177)	22.4 (477)	**0.003**	25.5 (655)
Antidepressant treatment if previous episode	91.0 (177)	77.1 (476)	**< 0.001**	80.9 (654)
Previous hospitalization for depression	15.3 (177)	6.5 (477)	**< 0.001**	8.9 (655)
Remission of previous episode	72.3 (177)	87.2 (477)	**< 0.001**	83.2 (655)
Previous suicide attempt	18.1 (177)	11.3 (477)	**0.023**	13.1 (655)

*Abbreviations: ASEX* Arizona Sexual Experience Scale, *CGI-S* Clinical Global Improvement Severity scale, *EQ-5D* EuroQol 5-Dimensions questionnaire, *MADRS* Montgomery–Åsberg Depression Rating Scale, *MCS* mental component summary, *PCS* physical component summary, *PHQ-9* 9-item Patient Health Questionnaire, *SD* standard deviation, *SDS* Sheehan Disability Scale, *SF-12* Medical Outcomes Study Short-Form (12-item) Health Survey, *WPAI* Work Productivity and Activity Impairment questionnaire

*P*-values in bold indicate statistically significant differences

^a^Number of patients included in the between-group comparison; information regarding whether the patient was initiating or switching treatment was missing for two patients

^b^Total number of patients providing data

^c^Physicians’ response

^d^The percentages refer to patients “probably” or “definitely” presenting clinically significant symptoms of anxiety according to physician; the *P*-value indicates the between-group comparison taking into account all five categories (“definitely”, “probably”, “probably not”, “definitely not” and “don’t know”)

^e^Assessment used by psychiatrists only

^f^Activity impairment due to problem

^g^UK sample population only

There were statistically significant demographic differences between the two patient groups: compared with patients who were initiating treatment, those who were switching were significantly older (*P* = 0.012) and more likely to be widowed or divorced/separated (Table 1). Patients who were initiating antidepressant treatment were more likely to be educated to university degree level and were more likely to be in paid employment or self-employed compared with patients who were switching antidepressant treatment (Table 1).

### Clinical profile

With regard to their current episode of depression, the duration of the episode – classified in time categories – differed significantly (*P* < 0.001) between the two patient groups. Greater proportions of patients who were switching antidepressants than those initiating treatment had been experiencing the current episode of depression for more than 8 weeks (Table 2). In addition, a significantly higher proportion of patients who were switching treatment were experiencing symptoms of anxiety (*P* < 0.001); among patients with anxiety, those who were switching were significantly more likely to be treated with anxiolytics (*P* = 0.004).

**Fig. 1:**
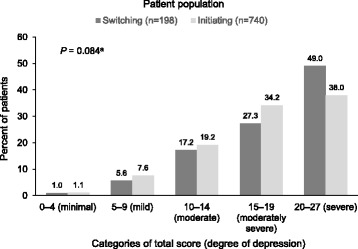
Distribution of PHQ-9 score categories at baseline, by treatment status. ^a^The between-group comparison of the categorical data was conducted using a chi-squared test. *Abbreviations: AD* antidepressant, *PHQ-9,* 9-item Patient Health Questionnaire

Severity of illness, as rated by the patient using the PHQ-9, indicated little clinical difference between the treatment groups. Although the between-group difference in mean scores reached statistical significance (*P* = 0.014), the mean score was only marginally higher for patients switching antidepressants versus those initiating treatment (Table 2). Conversely, the difference in distribution by category of severity did not reach statistical significance (*P* = 0.084), despite a greater proportion of patients who switched treatment ranking their illness as severe (PHQ-9 score 20–27) compared with those initiating antidepressant treatment (Fig. 1).

**Fig. 2:**
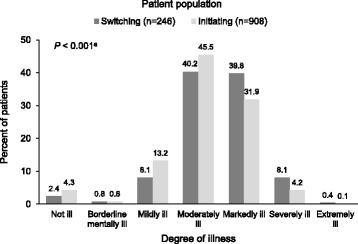
Distribution of CGI-S score categories at baseline by treatment status. ^a^The between-group comparison of the categorical data was conducted using Fisher’s exact test. *Abbreviations: AD* antidepressant, *CGI-S* Clinical Global Improvement Severity scale

The severity of the current depressive episode, as measured by the mean physician-assigned CGI-S score, was significantly higher in patients who were switching antidepressants compared with those initiating the treatment (*P* < 0.001) (Table 2). The profile of disease severity differed significantly in the two populations (*P* < 0.001), with a greater proportion of patients in the switching group rated as markedly, severely or extremely ill (48.4% versus 36.2% in the initiating group) (Fig. 2).

Compared with patients initiating antidepressant monotherapy, significantly greater proportions of patients switching antidepressants had experienced a previous episode of depression at any time (*P* < 0.001; Table 2).

No significant differences were seen with regard to the presence of comorbid mental disorders, but differences were noted for comorbid physical disorders: significantly higher proportions of patients who were switching had a diagnosis of a chronic pain (*P* < 0.001) or fibromyalgia (*P* < 0.001) compared with those initiating treatment (Table 2).

### Functioning and health-related quality of life

With regard to functioning as assessed by the SDS, the mean total SDS score was significantly higher – indicating greater functional impairment – in patients who were switching compared with those initiating antidepressants (*P* = 0.004) (Table 2). Furthermore, the individual SDS domain scores were also significantly higher in patients who were switching (each *P* < 0.05) (Table 2). The WPAI dimension of overall activity impairment showed a significant between-group difference, with a significantly higher mean score, i.e. worse impairment, in the group switching than in the group initiating antidepressants (*P* = 0.002; Table 2). The mean total ASEX sexual-function score was statistically significantly higher in the group switching than in the group initiating antidepressants (*P* = 0.031), although the numerical difference was small (Table 2).

The SF-12 mean physical component summary (PCS) score was significantly lower (indicating worse physical functioning) in the switching compared with the initiating group (*P* < 0.001); however the mean mental component summary (MCS) score did not differ between groups (Table 2).

### Resource use

Resource use within the 12 weeks before the baseline visit by patients with a depressive episode of greater than 8 weeks’ duration is summarized in Table 3. Compared with patients who initiated antidepressant medication, a statistically significantly greater proportion of patients who were switching antidepressants made at least one visit to any physician in the previous 12 weeks (88.0% vs 96.2%; *P* = 0.003). Patients who were switching antidepressants had more physician visits than patients initiating treatment in the same period (Table 3).

**Table 3:** Resource use for patients with a depressive episode of more than 8 weeks’ duration^a^

Resource use in the past 12 weeks	Percent of patients (*n* analysed)^b^		*P*-value: switching vs initiating	Percent of patients overall (*n*)^c^
	Switching	Initiating		
No. of physician visits	(157)	(432)	**< 0.001**	(589)
0	3.8	12.0		9.8
1	10.8	22.5		19.4
2	11.5	17.6		16.0
3	17.2	13.2		14.3
≥ 4	56.7	34.5		40.4
Unknown	0	0.2		0.2
General practitioner (any cause)^d^	91.2 (160)	82.8 (435)	**0.010**	85.0 (595)
Psychiatrist^d^	27.5 (160)	15.9 (435)	**0.001**	19.0 (595)
Psychotherapist/counselling^d^	19.4 (160)	12.9 (435)	**0.047**	14.6 (595)
Other specialist^d^	26.2 (160)	19.1 (435)	**0.057**	21.0 (595)
Hospitalization	3.8 (160)	2.1 (435)	0.248	2.5 (595)
Sick leave	90.5 (63)	78.0 (141)	**0.033**	81.9 (204)

*P*-values in bold indicate statistically significant differences

^a^*n* = 595 patients in total: *n* = 160 switching and *n* = 435 initiating antidepressant therapy

^b^Number of patients included in between-group comparison

^c^Total number of patients providing data

^d^Patients (*n* = 8–20 in total for each resource) for whom the information was reported as ‘unknown’ by the physician were included in the analysis as having used the resource

In terms of the type of physician visited, patients who were switching were also significantly more likely than those initiating treatment to have visited a psychiatrist (*P* = 0.001) or psychotherapist/counselling (*P* = 0.047) in the previous 12 weeks. A significantly greater proportion of patients switching antidepressants than those initiating treatment had taken at least one period of sick leave in the previous 12 weeks (*P* = 0.033).

## Discussion

The objective of this paper was to describe and compare the baseline characteristics of patients with MDD who initiated versus switched antidepressant treatment for the first time at inclusion in the PERFORM study. Overall, the baseline characteristics indicate significant differences between patients who are initiating and those who are switching antidepressants (i.e. undergoing first- and second-line monotherapy, respectively) in the course of a depressive episode. Compared with patients initiating antidepressants, those switching antidepressants tended to have more severe depressive symptoms, greater anxiety, worse health-related quality of life and greater functional impairment; be more likely to experience chronic pain and fibromyalgia; have a different socioeconomic and medical-history background; and use more healthcare resources.

Switching antidepressants is likely to be a common approach in clinical practice [10–13]. This is particularly the case in patients who fail to achieve an adequate response with an initial antidepressant [10, 13]. For example, in a retrospective cohort study of medical records of patients with depression in Spain, 43% of those with an inadequate response to first-line antidepressant treatment were switched to another antidepressant [13]. A high proportion of patients in clinical trials and clinical practice do not respond adequately to first-line treatment [2, 4, 26–32]. Switching antidepressants has been demonstrated to be effective in a number of clinical studies, with remission rates of up to 42% achieved in patients who previously failed to achieve remission [14, 15, 33]. While current guidelines recommend switching antidepressants if a patient who has adhered to the prescribed treatment regimen fails to achieve a satisfactory response to initial antidepressant treatment [5, 8], they do not generally recommend a specific assessment of severity, functioning or other aspects of the patient’s depressive episode when considering switching to second-line therapy.

The observation in the current study that the patients switching antidepressants were reporting more severe depressive symptoms than those initiating antidepressants is of particular note, given that the patients switching treatment had been receiving antidepressant treatment for some time. The patients who switched treatment may have been even more severely depressed when they initiated treatment. Although the mean between-group differences in depression severity scores were not large, they were consistent across patient (PHQ-9) and physician (CGI-S) reports.

Approximately 77% of patients who switched in the present study did so because of lack of efficacy. However, in other studies lower proportions of patients have cited this as the reason: a previous longitudinal naturalistic study found that 41% of patients with depression who switched within 4 weeks of initiating treatment said it was due lack of efficacy [34]. The relatively high proportion noted in the current study may in part be due to selection bias as discussed below.

Differences in the profile of the current depressive episode and in the history of psychiatric illness have previously been noted between patients who switch to second-line treatment and those who maintain first-line treatment [12]. In a study using historical cohort data from the UK’s GPRD, patients who switched antidepressant had more severe depression, were more likely to have concomitant anxiety disorders and were more likely to have experienced a previous episode of depression than those maintaining treatment [12]. The present study augments such findings by providing a more detailed clinical profile of switching and non-switching populations.

The results of the present analysis also suggest that patients who fail to respond adequately to first-line treatment may experience more severe functional impairment. In a cohort study Trivedi and colleagues [16] found that although patients who responded to first-line antidepressant treatment experienced improvements in work productivity in parallel with reductions in the severity of depressive symptoms, patients receiving second-line treatment (i.e. those who switched or received augmented treatment) did not experience such improvements when they responded to treatment.

Compared with maintaining treatment, switching has also been found to be associated with worse health-related quality-of-life outcomes in a longitudinal study of inpatients with MDD [35]. In the present study, the mean SF-12 PCS score in the group switching antidepressants was considerably lower than might be expected in the general population [23, 36, 37], which may also reflect the frequency of chronic pain and fibromyalgia in the study group. As would be expected, the mean MCS scores were far lower than in the general population (attributed a score of 50 ± 10.0 in a US general population and 48.9 ± 9.2 in a Greek general population) [23, 36, 37]. The mean MCS scores were similar in the two patient groups in the current study. This is surprising given the overall more severe depressive clinical profile in the group switching antidepressants; however, this may reflect the improved sensitivity expected from a disease-specific (PHQ-9) versus a generic instrument (SF-12).

In addition to factors such as those discussed above, there are a multitude of other aspects of a patient’s life that influence their recovery from MDD, including life events and personal circumstances. In the present study patients who switched medication had a longer duration of illness, were older, were more likely to be widowed or divorced, had lower levels of educational achievement and were more likely to be unemployed than patients initiating treatment. While it is possible to speculate on the role of such factors in patients’ response to treatment and recovery from MDD, their contribution is difficult to gauge. Social disadvantage, greater depression severity and anxiety have previously been linked to reduced likelihood of achieving remission after switching to second-line therapy in patients with MDD [38].

Previous studies have demonstrated increased healthcare resource use by patients who switch therapy compared with those who maintain their initial antidepressant therapy [13, 39, 40]. For example, using data from a US medical and pharmacy claims database, Schultz and Joish [39] found that patients with MDD who switched therapy incurred statistically significantly more depression-related ambulatory, emergency-room and inpatient visits than those who maintained their antidepressant therapy, leading to almost double the depression-related costs. The greater resource use observed in patients who switch would be anticipated, given their requirement for treatment before switching.

Depression-associated functional impairment and reductions in health-related quality of life have previously been shown to be linked to the severity of the illness [41, 42]. Some functional comorbidities, such as chronic pain and fibromyalgia – which were significantly more prevalent in the group switching antidepressants in the present study – may influence health-related quality of life independently of depressive symptoms and may contribute to greater severity of depression and functional impairment [43–46]. Indeed, there is evidence of complex interrelationships between depression and pain [47, 48]. The greater prevalence of anxiety in the group switching antidepressants may also contribute to the greater severity of depression seen in this group [49].

As in other therapeutic areas, with greater understanding of the underlying pathophysiology of depression and of the mechanisms of action of antidepressants, there is the potential for personalized treatment, such that patients with particular depressive symptoms or features may be prescribed specific pharmacotherapies [50]. Careful consideration of the patient’s full profile with the aim of optimizing antidepressant treatment as early as possible is warranted in light of observations that the risk of treatment failure increases with the number of lines of treatment required [2].

### Strengths and limitations

The strengths of this study include the large sample size and its international scope. In addition, this is one of the first studies in which the collected baseline data comprise such a wealth of naturalistic information, including detailed individual medical histories and both physician- and patient-reported information on the current depressive episode, as well as functional, quality-of-life and resource-use data. The study population had the expected demographic characteristics of patients with depression: as previously reported in other observational studies, the majority of patients with depression were women, and the mean age of the population was approximately 45 years [11, 51, 52]. Although the categorization of depression severity using patient-reported outcome in the majority of patients in the present study (rather than by scales more frequently used in clinical trials) may be perceived as a limitation of the study, this approach is increasingly recognised as appropriate and may be considered a study strength. Greater focus on patient-reported outcomes reflects the general move towards increased patient involvement in treatment decisions, and awareness of the limitations of the more ‘traditional’ clinical symptom-based measures in assessing recovery from mental illness in a way that is meaningful to patients [53–56]. In PERFORM a validated questionnaire, based on DSM-IV diagnostic criteria (PHQ-9) [17] was used to evaluate patient-reported depression, and the severity levels revealed by physician assessment using the CGI-S support the findings of the patient-reported assessment that patients switching antidepressants had more severe depression.

We acknowledge a number of study limitations. Patients were selected on the basis of having symptoms of depression, resulting in an over-representation in the switching population of patients switching treatment due to lack of efficacy (i.e. inadequate response to the previous antidepressant) compared with other reasons. The difference in depression severity between the two study populations may therefore be greater than would be seen in clinical practice; however, as drug-related adverse effects (the next most common reason for switching) may affect functioning and quality of life to a similar degree as a lack of efficacy, the potential bias towards selecting patients with more severe depression in the switching group may not have an effect on the findings. The study comprised outpatients only, potentially excluding some of the most severely affected patients. These criteria may have led to the study population of patients switching treatment having less severe depression than would be encountered in patients switching treatment in clinical practice, however, in Europe the vast majority of patients with MDD are treated in the outpatient setting [1, 57]. The study also excluded patients treated with combination or augmentation therapies. However, guidelines currently applied in Europe recommend initiating treatment with antidepressant monotherapy, with a switch to a different monotherapy in the event that an inadequate response is achieved with the initial therapy [8]. It would therefore be anticipated that in clinical practice few patients would receive a combination of antidepressants or antidepressant therapy augmented with another agent as either first- or second-line therapy. Because of these criteria, the outcomes may be more applicable to patients in the early rather than later period of their depressive episode. A degree of recall bias may also have occurred, which would have resulted in more extensive retrospective reporting of medical issues and resource use by the patients switching treatment. However, such patients would genuinely have experienced more clinical encounters as they had been treated for depression in the preceding weeks; as the recall period was restricted to 12 weeks, the potential for recall bias is low. Analyses of the MADRS and EQ-5D data were based on small sample numbers as these tools were only used in specific settings. This limits the statistical power of these analyses to detect between-group differences. However, the reasonably good response rate for other assessments of depression severity and quality of life – despite the fact that completion of the self-administered instruments was on an entirely voluntary basis – permitted reliable comparisons for these characteristics.

## Conclusions

In conclusion, the results of this analysis suggest that patients with depression who are switching treatment have different profiles and depression-associated health needs and should be managed differently from patients who are initiating treatment. In such cases the physician should consider the severity of depression and other aspects of the patient’s illness, such as daily functioning and anxiety, in order to select the optimal second-line treatment.

## Additional files

Additional file 1: List of participating ethics committees. Details of all participating local ethics committees. (DOCX 18 kb)

## Abbreviations

ASEX: Arizona Sexual Experience Scale
CGI-S: Clinical Global Impressions–Severity of illness scale
DSM-IV-TR: Diagnostic and Statistical Manual of Mental Disorders IV Text Revision
EQ-5D: EuroQol 5-Dimensions questionnaire
GPRD: General Practice Research Database
MADRS: Montgomery–Åsberg Depression Rating Scale
MCS: Mental component summary
MDD: Major depressive disorder
PCS: Physical component summary
PERFORM: Prospective Epidemiological Research on Functioning Outcomes Related to Major depressive disorder
PHQ-9: 9-item Patient Health Questionnaire
SDS: Sheehan Disability Scale
SF-12: Medical Outcomes Study Short-Form (12-item) Health Survey
STAR*D: Sequenced Treatment Alternatives to Relieve Depression
WPAI: Work Productivity and Activity Impairment questionnaire
