# Supplementary material for: Characteristics of patients with depression initiating or switching antidepressant treatment: baseline analyses of the PERFORM cohort study
Source: BMC Psychiatry. 2018 Mar 27;18:80. doi: 10.1186/s12888-018-1657-3 (PMC5870247; doi:10.1186/s12888-018-1657-3)
Supplement: Supplementary file 1 — List of participating ethics committees. Details of all participating local ethics committees. (DOCX 18 kb) [file 12888_2018_1657_MOESM1_ESM.docx]

**Characteristics of patients with depression initiating or switching antidepressant treatment: baseline analyses of the PERFORM cohort study**

**Additional file 1: List of participating ethics committees**

**France**

Agence Nationale de Sécurité du Médicament et des produits de santé (ANSM; previously known as Agence Française de Sécurité Sanitaire des Produits de Santé [AFSSAPS]); Le Comité consultatif sur le traitement de l'information en matière de recherche (CCTIRS); Commission nationale de l'informatique et des libertés (CNIL); Le Conseil de l'ordre des médecins (CNOM); and Comité de Protection des Personnes (CPP), Ile de France II.

**Germany**

Munich Ethics Committee; Ethics Committees of the Medical Associations of: Baden-Württemberg, Bavaria, Hamburg, Hessen, Niedersachsen, Nordrhein, Rheinland-Pfalz, Saarland, Sachsen, Sachsen-Anhalt, Thüringen, Westfalen-Lippe, and Berlin.

**Spain**

Agencia Española del Medicamento y Productos Sanitarios (AEMPS); Comunidades Autónomas (CCAA) of Andalucia, Aragón, Baleares, Castilla La Mancha, Castilla y León, Cataluña, Comunitat Valenciana, Extremadura, Galicia, Murcia, Navarra, and País Vasco; Comités Eticos de Investigaciones Clinicas (CEIC): Baleares (CS Santa Ponça), Castilla la Mancha (Hospital General Ciudad Real), Navarra (CEIC Clinica Universitaria Navarra), Extremadura (Servicio de Psiquiatria Complejo, Hospital Caceres), Murcia (Centro de Salud, Jumilla Hospital Virgen del Castillo), Andalucia (Centro Hospital Universitario Virgen Macarena), Pais Vasco (Centro de Salud Repelega [Portugalete]), Valencia (Hospital Universitario de Sant Joan D'alacant, Unidades de Conductas Adictivas 19 Alicante, Hospital Virgen De Los Lirios: Servicio de Psiquiatria) and Cataluña (Comité Etic d'Investigacio Clinica. Institut Universitari d'Investigació en Atenció Primària [IDIAP] Jordi Gol Barcelona).

**Sweden**

Uméå Ethics Committee

**United Kingdom**

Medical Research and Ethics Committee (MREC; Brighton and Hove City Primary Care Trust), National Institute for Health Research (NIHR); and the Primary Care Trusts of: Bath, Brighton & East Sussex, Cardiff and Vale, Cornwall and Isles of Scilly, Croydon, Derbyshire, Devon, Dorset, Hampshire, NHS Hertfordshire, Hounslow, Islington, NHS Lancashire, NHS London, NHS Norfolk, Nottingham, NHS Manchester, Oldham, Redcar and Cleveland, Sheffield, Surrey, NHS Warwickshire, West Kent, West Sussex, Wiltshire, Wirral, Yorkshire and North York.
